# Does feeding starch contribute to the risk of systemic inflammation in dairy cattle?

**DOI:** 10.3168/jdsc.2022-0303

**Published:** 2022-12-01

**Authors:** K.C. Krogstad, B.J. Bradford

**Affiliations:** Department of Animal Science, Michigan State University, East Lansing 48824

## Abstract

In the high-producing dairy cow, providing an adequate supply of digestible energy is essential. One strategy to meet this need is to provide fermentable starch from cereal grains or silages like corn, barley, or wheat. Unfortunately, excess dietary starch increases the risk of rumen acidosis. Rumen acidosis challenge models using high-grain diets, particularly with wheat and barley, have demonstrated that a sudden change in starch concentration or digestibility leads to the breakdown of the rumen epithelial barrier. As a result, increases in circulating lipopolysaccharide (a marker of bacterial translocation) and acute phase proteins (APP) have been observed. Feeding increasing amounts of starch in chronic feeding studies does not appear to consistently modulate inflammation in early-lactation cows that already experience inflammation. In mid- and late-lactation cows, increasing starch above 30% may increase APP, but the response is inconsistent and has not been investigated using different grains or differently processed starch sources. Abomasal starch infusion experiments indicate that increasing the intestinal starch supply consistently reduces fecal pH but does not lead to an APP response or changes in gut integrity. Increasing intestinal starch supply increases fecal butyrate concentrations, and butyrate has had positive effects on gut health and integrity in other species and experimental models. More chronic feeding experiments are needed to investigate how starch concentrations, sources, processing methods, and interactions affect inflammation and gut integrity. There is a paucity of data investigating the role that carbohydrate concentrations and sources play on ruminant hindgut health, integrity, function, structure, or microbiome. Currently, data indicate that feeding diets with less than 30% starch to lactating dairy cows does not contribute to systemic inflammation.

## Highlights

• Barley and wheat grain acidosis challenges result in systemic inflammation.
• Abomasally infusing starch does not appear to cause inflammation.
• Abrupt diet change may contribute to inflammation when increasing dietary starch.
• Increasing starch fed to postpartum cows does not consistently alter inflammation.

## Graphical Abstract

**Figure:**
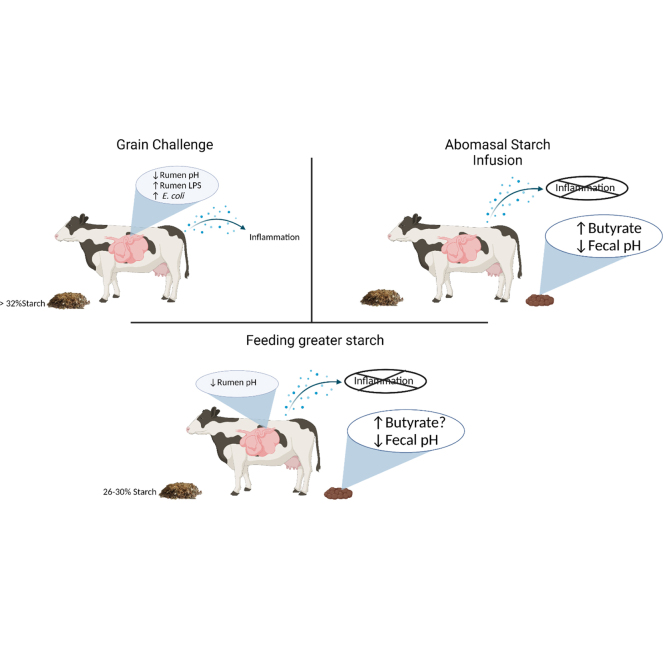
**Summary:** Grain challenge experiments, where a portion of the diet is suddenly replaced by highly fermentable grains, result in systemic inflammation. Both high starch fermentability and sudden diet changes likely play a part in causing this inflammation. Abomasally infusing starch does not seem to result in inflammation but it does consistently reduce fecal pH and increase fecal butyrate concentrations. Direct effects of starch on the hindgut epithelial barrier and mucosal layers are unknown in dairy cattle. Increasing starch concentration in dairy cow rations does not consistently result in inflammation even though it reduces rumen and fecal pH. Research investigating the effects of different grain sources and processing methods on systemic inflammation is warranted.

Starch and the cereal grains that typically supply it play an important role in dairy cattle nutrition. Starch is a highly digestible energy source that may increase milk yield (Sánchez-Duarte et al., 2019), milk protein yield (Dias et al., 2018; McCaughern et al., 2020; Morris et al., 2020), and body tissue accretion (Boerman et al., 2015) compared with feeding NDF or specific fatty acids. Furthermore, increasing the concentration of starch from ~20% to ≥28% of diet DM reduces ruminal (Salfer et al., 2018) and hindgut (Neubauer et al., 2020) pH. Reducing digesta pH may be one factor contributing to diminished gut health and integrity. Increased luminal LPS concentration (Li et al., 2012, 2016) and osmolality (Chibisa et al., 2016) when feeding starch may also be contributing factors.

This mini-review summarizes current research investigating systemic inflammation in lactating dairy cows provided different dietary starch concentrations during grain challenges, feeding experiments, or hindgut infusions. We use “grain challenge” when referring to experiments where grains are abruptly increased to ≥20% of diet DM for the purpose of causing ruminal acidosis. These grain challenges have resulted in an increase in circulating markers of inflammation such as LPS and acute phase proteins (**APP**; Khafipour et al., 2009b). A meta-analysis evaluating acidosis grain challenge data indicated that >44% concentrates or <39% NDF in diets fed to dairy cattle increases the risk of inflammation and increased circulating APP (Zebeli et al., 2012). Furthermore, ruminal acidosis grain challenges break down the rumen epithelial barrier (Steele et al., 2011), which may lead to an inflammatory response due to rumen bacteria or their components moving across the epithelium and interacting with circulating and tissue-resident immune cells. Inflammation may also result from free ruminal LPS binding its receptor, toll-like receptor 4 (TLR4), on the rumen epithelium, which has been shown to increase mRNA abundance in rumen tissue during acidosis (Pederzolli et al., 2018). Either may lead to the release of proinflammatory cytokines and eventual increases in circulating APP, but consensus on the mode of action is lacking.

Experiments that involve feeding elevated starch concentrations, usually ≥28% of diet DM as starch, and measuring its effects on inflammation have increased in the last 2 decades. Investigators have observed increases in plasma LPS, serum amyloid A (**SAA**), haptoglobin (**Hp**), and LPS binding protein during these rumen acidosis grain challenge experiments (Gozho et al., 2007; Khafipour et al., 2009b; Li et al., 2016). One experiment reported increases in serum Hp from nondetectable concentrations to 600 µg/mL in grain-challenged cows (Khafipour et al., 2009c). Interestingly, feeding pelleted alfalfa hay induced similar reductions in rumen pH and increases in ruminal LPS and VFA, but did not increase plasma LPS or APP (Khafipour et al., 2009a,c), which indicates that inflammation likely does not stem from reduced rumen pH and increased rumen LPS concentration alone. Other factors such as diet adaptation or gut microbiome may play a role in this diet-induced inflammation (Plaizier et al., 2017). We do not know whether the APP responses observed during acidosis challenge studies originate from the rumen or intestine, although the rumen epithelium clearly degrades during severe ruminal acidosis (41% dietary starch; Steele et al., 2011). Abeyta et al. (2022) also observed increased APP in plasma during a severe acidosis challenge (2.75% of BW as corn; ~21 kg). When rumen fluid from these grain-challenged donor cows was abomasally infused into nonchallenged recipients, they did not observe an increase in APP.

Data suggest that rumen adaptation plays a role in the APP response. In the non-grain-challenge model (Khafipour et al., 2009a,c), where alfalfa hay was gradually replaced by pelleted alfalfa hay over 6 wk (22% dietary starch), there was no systemic inflammatory response, whereas abrupt diet change in the grain challenge experiments led to an APP response (Khafipour et al., 2009b). Gott et al. (2015) found that a high-starch diet with corn grain (~29% starch) did not increase SAA, whereas an acidosis induction diet that provided corn and wheat grain (~32% starch) suddenly fed for 2 d after cows had been on a control diet (~24% starch) resulted in increased SAA. Sudden dietary changes may be more to blame for the APP response than chronically elevated dietary starch. Others have also observed no significant difference or reductions in circulating Hp when increasing dietary starch (Table 1; Ertl et al., 2015; Dias et al., 2018; McCaughern et al., 2020; Haisan et al., 2021).

**Table 1:** Experiments where lactating dairy cows were fed different dietary starch concentrations in chronic feeding regimens and inflammatory biomarkers were assessed

Study	Starch source	Starch concentration	DIM^1^	Key responses to additional starch^2^
Albornoz et al. (2020)	Corn	22%, 28%	1	↑ RONS when fed high-moisture corn
Dias et al. (2018)	Corn	23%, 29%	176 ± 18	↔ Hp ↓ Rumen pH ↑ Milk protein yield
Emmanuel et al. (2008)	Barley	NR^3^ (35%, 38%, 42%, 45% NFC)	60–140	↑ Rumen LPS ↑ SAA
Ertl et al. (2015)	Corn, wheat, oats	7%, 12%	108 ± 90	↔ Hp
Gott et al. (2015)	Corn, wheat	24%, 28%, 32%	140 ± 16	↑ SAA when fed acidosis induction diet ↔ SAA when fed high-starch diet
Haisan et al. (2021)	Corn, barley	25%, 32%	−28 ± 3	↓ Hp and SAA
McCarthy et al. (2015b)	Corn	21%, 26%	1	↑ Hp ↓ NEFA and BHB
McCaughern et al. (2020)	Corn, wheat	15%, 22%	33 ± 2.5	↔ Hp ↑ Ceruloplasmin ↓ Rumen pH ↑ Milk protein yield
Tayyab et al. (2022)	Corn, wheat	9%, 22%, 22%, 32%	61 ± 0.2	↑ Hp ↔ Mean rumen pH

1 DIM at the start of the study.

2 NEFA = nonesterified fatty acid; RONS = reactive oxygen and nitrogen species; SAA = serum amyloid A; Hp = haptoglobin; ↔ = no change, ↑ = significant increase, ↓ = significant decrease.

3 NR = not reported.

Increasing dietary starch reduces measurements of bacterial diversity (Deusch et al., 2017; Plaizier et al., 2017; Neubauer et al., 2020). Also, grain versus pelleted alfalfa acidosis induction results in different microbial population shifts. Khafipour et al. (2009c) investigated the microbial population in the rumen of dairy cows who were subject to either grain or alfalfa acidosis challenges. They observed a 64-fold increase in ruminal *Escherichia coli* during the grain challenge experiment. They also discovered that ruminal *E. coli* concentrations were the greatest predictor of severity of acidosis, which was determined by serum Hp, rumen pH, and free rumen LPS. Grain-based acidosis shifted the *E. coli* populations to strains with greater virulence compared with that induced by alfalfa-based challenges (Khafipour et al., 2011). The genes associated with virulence were related to adhesion of *E. coli* to epithelial cells. Increases in strains of *E. coli* with greater adhesion capabilities during grain-based acidosis challenges may be related to the increased inflammation observed in grain versus non-grain challenges. Increased adhesion capability is problematic during acidosis because nonkeratinized cells that are exposed to rumen contents are more susceptible to adhesion by microbes like *E. coli* (Gálfi et al., 1998). By increasing both *E. coli* and the sloughing of the stratum corneum during acidosis (Steele et al., 2011), grain-based acidosis challenges may present more risk of inflammation compared with chronic feeding models.

A more thorough investigation of the digestive tract microbiome was conducted by Plaizier et al. (2017). They observed increased *E. coli* in cecal and fecal contents of cows subjected to grain acidosis challenges. It seems that increasing starch in the rumen or its flow to the hindgut may increase problematic microbial populations, such as *E. coli.* This alteration in gut bacteria may increase the risk of inflammation when feeding high-starch diets, but investigations in a greater diversity of diets are warranted.

Increases in APP may occur when starch concentrations are ≥30% of diet DM and when barley is the primary grain being fed. Late-lactation cows fed 32% starch diets (barley grain and barley silage as starch sources) had elevated serum Hp (1,500 µg/mL). Peak-lactation cows fed 45% of diet DM as barley grain (45% NFC, 25% NDF, 30% forage) had reduced rumen pH, increased ruminal LPS, and increased SAA, but milk yield increased by 4 kg/d nonetheless, whereas milk fat yield was reduced (Emmanuel et al., 2008; Zebeli and Ametaj, 2009). Pan et al. (2017) increased dietary starch from 20 to 33% of diet DM by increasing corn grain and observed reduced rumen pH and a 10-fold increase in ruminal LPS. Plasma IL-1β and IL-6 increased by 50 and 20%, respectively. Rumen epithelial protein abundance of TLR4 and proinflammatory cytokines was increased, pointing to the possibility that the rumen was the source of increased cytokines in plasma. Pan et al. (2017), similar to Emmanuel et al. (2008), observed increased milk yield although component yields were similar, because component concentrations were reduced in response to increasing dietary starch. Studies investigating the interactions of starch concentrations with starch source (i.e., corn) or processing methods (i.e., ensiling, steam flaking, cracking, rolling) are needed because the most data stem from wheat- or barley-based diets.

Few studies have examined periparturient cow APP responses to increasing dietary starch concentrations, but there is little evidence of enhanced inflammation. Knoblock et al. (2019) observed similar APP concentrations in postpartum cows fed 22 or 28% starch diets. McCarthy et al. (2015b) fed 21 or 26% starch diets, and greater starch increased plasma Hp from 700 to 1,000 µg/mL. Increasing starch also increased plasma glucose and insulin and reduced plasma nonesterified fatty acid and BHB concentrations. Increases in monocyte function (Yasui et al., 2016) and improved energy balance (McCarthy et al., 2015a) may indicate improved health and resilience of the transition dairy cow fed additional starch. Albornoz et al. (2020) investigated both starch concentration (22 vs. 28%) and starch processing method (dry ground vs. high moisture) and observed greater reactive oxygen and nitrogen species when feeding high-moisture corn. They also observed increased Hp when increasing dietary starch in the high-moisture corn diets, which may indicate that enhancing rumen fermentability when feeding greater concentrations of starch is problematic. In contrast, Haisan et al. (2021) found that high-starch diets (32%) postpartum resulted in lower Hp and SAA. Feeding greater dietary starch to transition dairy cows does not consistently alter APP (Figure 1).

**Figure 1:**
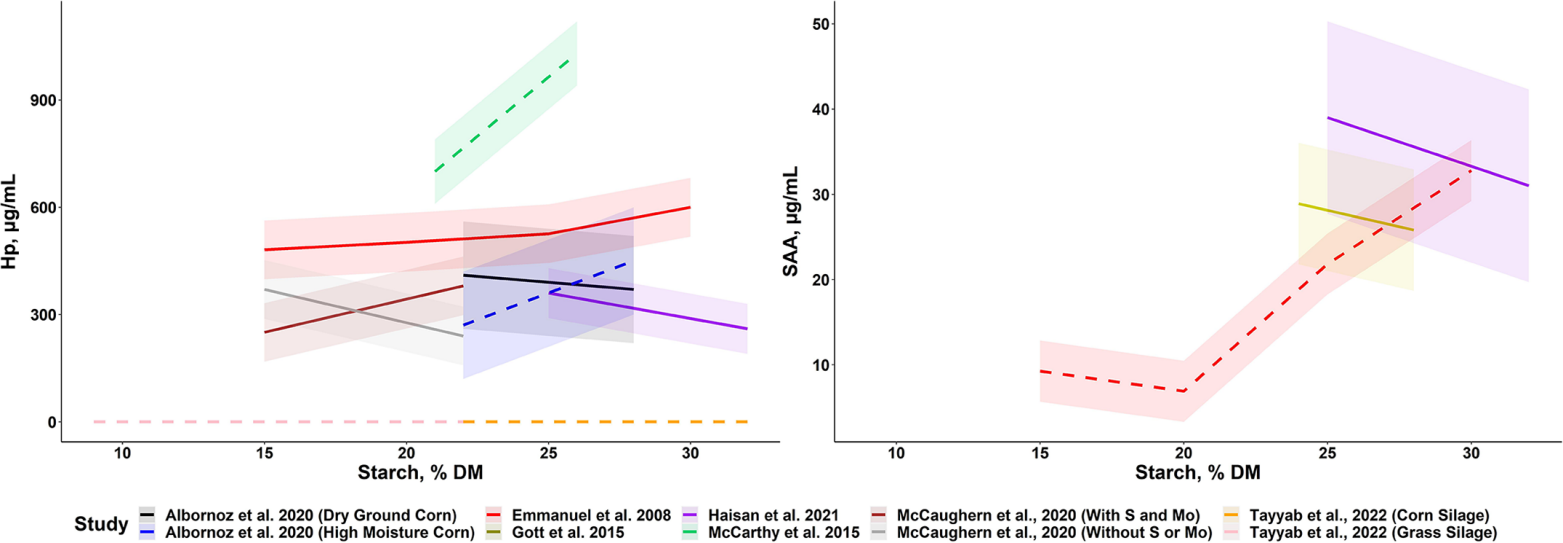
Summary of plasma haptoglobin (Hp) and serum amyloid A (SAA) means reported in chronic starch feeding experiments where lactating dairy cows were fed varying concentrations of dietary starch. Dashed lines indicate statistical significance detected in the experiment, whereas solid lines represent insignificant effects of starch concentration. Outliers for Hp (>1.5 μg/mL) were excluded from the figure (1 study). The Albornoz et al. (2020), Haisan et al. (2021), and McCarthy et al. (2015b) publications reported experiments using periparturient dairy cows; others used cows ranging from 30 to 150 DIM.

Abomasally infusing starch has not resulted in inflammation or gut barrier integrity loss even though it reduces fecal pH. Abeyta et al. (2019a) and Abeyta et al. (2019b) infused 4 kg/d of starch into the abomasum and fecal pH decreased from 6.8 to 5.9 and from 7.2 to 5.8, respectively. van Gastelen et al. (2021a) and van Gastelen et al. (2021b) infused up 3 kg of corn starch and 3 kg of ground corn, respectively, directly into the abomasum and fecal pH declined from 6.5 to 5.15 and from 6.8 to 6.0. Even though fecal pH was reduced, SAA and Hp were unchanged as a result of starch infusion. van Gastelen et al. (2021a) infused cows with Co-EDTA (an indigestible marker to estimate gut permeability) and did not observe any changes in gut permeability. These data indicate that increasing intestinal starch supply, independent of other factors, does not result in systemic inflammation.

Infusing starch abomasally dramatically increased fecal butyrate (van Gastelen et al., 2021a,b). Ruminal butyrate concentrations increase during ruminal acidosis as well (Khafipour et al., 2009b). Butyrate is an endogenous ligand for the hydroxycarboxylic acid receptor 2 (HCA2), which is involved in inflammatory signaling and tight junction formation (Plöger et al., 2012; Li et al., 2021). Butyrate produced during hindgut starch fermentation may provide benefits to the gut (Figure 2). Butyrate increased mRNA abundance of *IL10* (encoding an anti-inflammatory cytokine) in intestinal dendritic cells and macrophages, which, when incubated with naive CD4^+^CD25^−^ T cells, promoted development of regulatory T cells, which suppress inflammatory signaling (Singh et al., 2014). Butyrate also increased IL-18 secretion in colon epithelial cells (Singh et al., 2014), which supports a cell-mediated T-cell response that may promote inflammation. Butyrate modulates intestinal barrier function and integrity by increasing tight junction protein expression (Peng et al., 2009; Yan and Ajuwon, 2017). Supplementing butyrate to calves promotes rumen and hindgut development (Górka et al., 2018), but the authors acknowledge that little is known about the role of butyrate in the ruminant hindgut.

**Figure 2:**
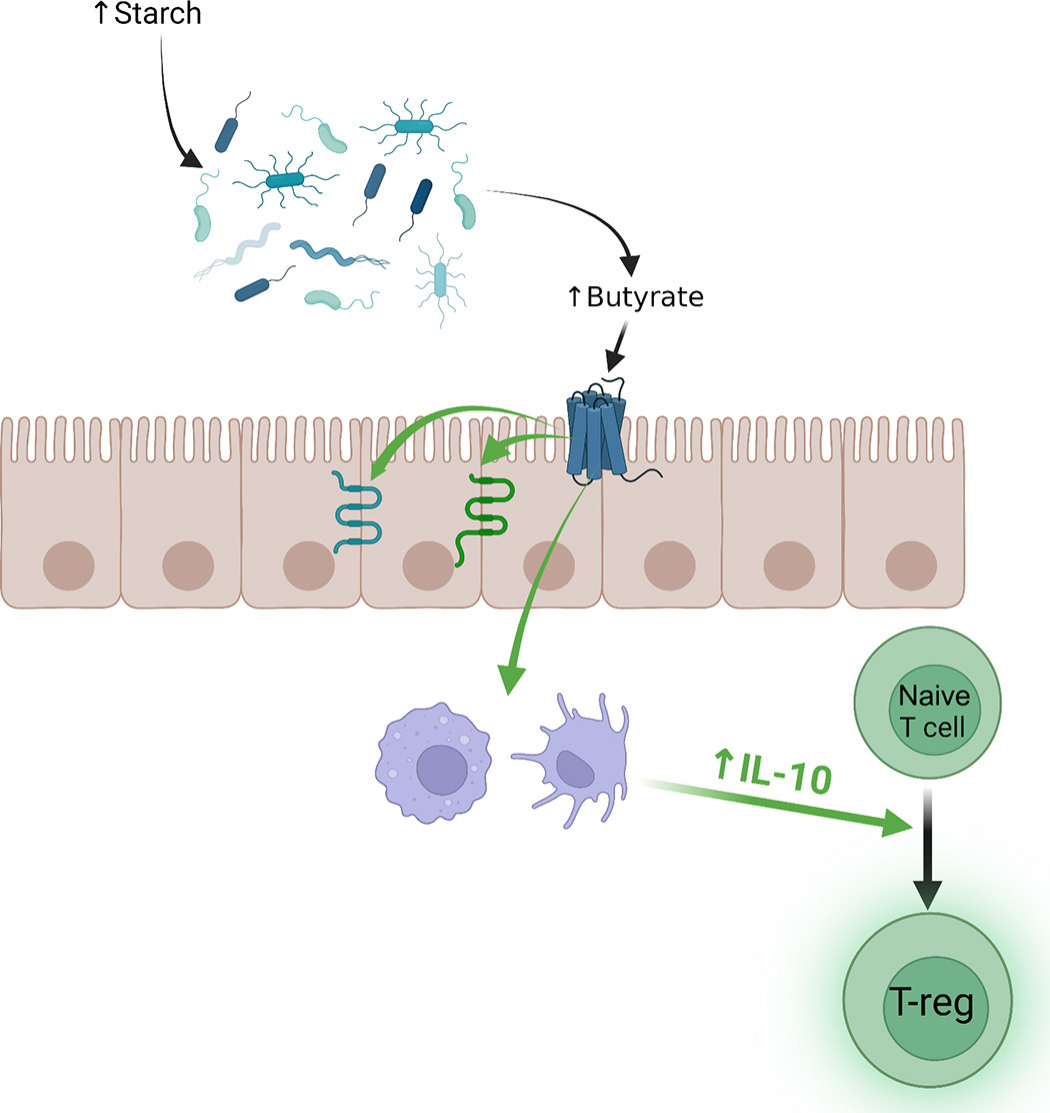
Possible effects of butyrate in the hindgut of dairy cows resulting from increased starch supply to the large intestine. Butyrate can increase tight junction mRNA and protein abundance in model species and cells. It also promotes IL-10 expression (anti-inflammatory) in dendritic cells and macrophages, which helps to promote T-regulatory cell development (inflammation-suppressing cell type). These effects may help to mitigate the risk of intestinal inflammation.

These data are compelling but there are gaps in our understanding of the effects of starch on gut health. For example, increased fecal butyrate indicates that butyrate concentration in the distal colon is increased, but we have yet to determine whether butyrate is increased in other areas of the hindgut. Furthermore, feeding additional starch may affect mucosal barriers that provide further protection for the hindgut tissue (Kim and Ho, 2010; Steele et al., 2016), but this has not been investigated in dairy cattle.

The potential effects of increasing dietary starch on systemic inflammation has been widely discussed in the dairy industry. During grain challenges in which diets are suddenly changed and forages are replaced by highly fermentable grains, acidosis and systemic inflammation occur. Extremely high concentrations of starch (≥30% of diet DM) in barley- or wheat-based diets may also lead to increased APP, but data indicate that sudden diet changes contribute to inflammation more than chronically elevated dietary starch. Altering dietary starch (20–32% of diet DM) for transition cows does not consistently affect APP. Starch infusion experiments demonstrate that increasing the supply of starch in the small intestine does not cause inflammation, even though fecal pH is reduced. Increasing intestinal starch supply increases fecal butyrate concentrations, which may provide benefits in the gut, but further investigation is needed. Evaluation of the gut microbiome and hindgut mucosal layers may yield important insights into the effects of starch on gut health and inflammation.

## References

[bib1] Abeyta M.A., Goetz B.M., Mayorga E.J., Rodriguez-Jimenez S., Ophenorth J., Freestone A.D., Lourenco M., Callaway T.R., Baumgard L.H. (2022). Effects of abomasally infused rumen fluid from corn-challenged donor cows on production, metabolism, and inflammation biomarkers in naïve recipient cows. J. Dairy Sci..

[bib2] Abeyta M.A., Horst E.A., Mayorga E.J., Goetz B.M., AlQaisi M., McCarthy C.S., O'Neil M.R., Dooley B.C., Piantoni P., Schroeder G.F., Ramirez-Ramirez H.A., Baumgard L.H. (2019). Effects of hindgut acidosis on metabolism, inflammation and production in dairy cows consuming a standard lactation diet. J. Dairy Sci..

[bib3] Abeyta M.A., Horst E.A., Rodriguez-Jimenez S.J., Mayorga E.J., Goetz B.M., AlQaisi M., Piantoni P., Schroeder G.F., Ramirez-Ramirez H.A., Baumgard L.H. (2019). Effects of hindgut acidosis on metabolism, inflammation, and production in dairy cows acclimated to a low-starch diet. J. Dairy Sci..

[bib4] Albornoz R.I., Sordillo L.M., Contreras G.A., Nelli R., Mamedova L.K., Bradford B.J., Allen M.S. (2020). Diet starch concentration and starch fermentability affect markers of inflammatory response and oxidant status in dairy cows during the early postpartum period. J. Dairy Sci..

[bib5] Boerman J.P., Potts S.B., VandeHaar M.J., Lock A.L. (2015). Effects of partly replacing dietary starch with fiber and fat on milk production and energy partitioning. J. Dairy Sci..

[bib6] Chibisa G.E., Beauchemin K.A., Penner G.B. (2016). Relative contribution of ruminal buffering systems to pH regulation in feedlot cattle fed either low- or high-forage diets. Animal.

[bib7] Deusch S., Camarinha-Silva A., Conrad J., Beifuss U., Rodehutscord M., Seifert J. (2017). A structural and functional elucidation of the rumen microbiome influenced by various diets and microenvironments. Front. Microbiol..

[bib8] Dias A.L.G., Freitas J.A., Micai B., Azevedo R.A., Greco L.F., Santos J.E.P. (2018). Effect of supplemental yeast culture and dietary starch content on rumen fermentation and digestion in dairy cows. J. Dairy Sci..

[bib9] Emmanuel D.G.V., Dunn S.M., Ametaj B.N. (2008). Feeding high proportions of barley grain stimulates an inflammatory response in dairy cows. J. Dairy Sci..

[bib10] Ertl P., Zebeli Q., Zollitsch W., Knaus W. (2015). Feeding of by-products completely replaced cereals and pulses in dairy cows and enhanced edible feed conversion ratio. J. Dairy Sci..

[bib11] Gálfi P., Neogrády S., Semjén G., Bardocz S., Pusztai A. (1998). Attachment of different *Escherichia coli* strains to cultured rumen epithelial cells. Vet. Microbiol..

[bib12] Górka P., Kowalski Z.M., Zabielski R., Guilloteau P. (2018). Invited review: Use of butyrate to promote gastrointestinal tract development in calves. J. Dairy Sci..

[bib13] Gott P.N., Hogan J.S., Weiss W.P. (2015). Effects of various starch feeding regimens on responses of dairy cows to intramammary lipopolysaccharide infusion. J. Dairy Sci..

[bib14] Gozho G.N., Krause D.O., Plaizier J.C. (2007). Ruminal lipopolysaccharide concentration and inflammatory response during grain-induced subacute ruminal acidosis in dairy cows. J. Dairy Sci..

[bib15] Haisan J., Inabu Y., Shi W., Oba M. (2021). Effects of pre- and postpartum dietary starch content on productivity, plasma energy metabolites, and serum inflammation indicators of dairy cows. J. Dairy Sci..

[bib16] Khafipour E., Krause D.O., Plaizier J.C. (2009). Alfalfa pellet-induced subacute ruminal acidosis in dairy cows increases bacterial endotoxin in the rumen without causing inflammation. J. Dairy Sci..

[bib17] Khafipour E., Krause D.O., Plaizier J.C. (2009). A grain-based subacute ruminal acidosis challenge causes translocation of lipopolysaccharide and triggers inflammation. J. Dairy Sci..

[bib18] Khafipour E., Li S., Plaizier J.C., Krause D.O. (2009). Rumen microbiome composition determined using two nutritional models of subacute ruminal acidosis. Appl. Environ. Microbiol..

[bib19] Khafipour E., Plaizier J.C., Aikman P.C., Krause D.O. (2011). Population structure of rumen *Escherichia coli* associated with subacute ruminal acidosis (SARA) in dairy cattle. J. Dairy Sci..

[bib20] Kim Y.S., Ho S.B. (2010). Intestinal goblet cells and mucins in health and disease: Recent insights and progress. Curr. Gastroenterol. Rep..

[bib21] Knoblock C.E., Shi W., Yoon I., Oba M. (2019). Effects of supplementing a *Saccharomyces cerevisiae* fermentation product during the periparturient period on the immune response of dairy cows fed fresh diets differing in starch content. J. Dairy Sci..

[bib22] Li S., Khafipour E., Krause D.O., Kroeker A., Rodriguez-Lecompte J.C., Gozho G.N., Plaizier J.C. (2012). Effects of subacute ruminal acidosis challenges on fermentation and endotoxins in the rumen and hindgut of dairy cows. J. Dairy Sci..

[bib23] Li S., Yoon I., Scott M., Khafipour E., Plaizier J.C. (2016). Impact of *Saccharomyces cerevisiae* fermentation product and subacute ruminal acidosis on production, inflammation, and fermentation in the rumen and hindgut of dairy cows. Anim. Feed Sci. Technol..

[bib24] Li Z., McCafferty K.J., Judd R.L. (2021). Role of HCA2 in regulating intestinal homeostasis and suppressing colon carcinogenesis. Front. Immunol..

[bib25] McCarthy M.M., Yasui T., Ryan C.M., Mechor G.D., Overton T.R. (2015). Performance of early-lactation dairy cows as affected by dietary starch and monensin supplementation. J. Dairy Sci..

[bib26] McCarthy M.M., Yasui T., Ryan C.M., Pelton S.H., Mechor G.D., Overton T.R. (2015). Metabolism of early-lactation dairy cows as affected by dietary starch and monensin supplementation. J. Dairy Sci..

[bib27] McCaughern J.H., Mackenzie A.M., Sinclair L.A. (2020). Dietary starch concentration alters reticular pH, hepatic copper concentration, and performance in lactating Holstein-Friesian dairy cows receiving added dietary sulfur and molybdenum. J. Dairy Sci..

[bib28] Morris D.L., Brown-Brandl T.M., Hales K.E., Harvatine K.J., Kononoff P.J. (2020). Effects of high-starch or high-fat diets formulated to be isoenergetic on energy and nitrogen partitioning and utilization in lactating Jersey cows. J. Dairy Sci..

[bib29] Neubauer V., Petri R.M., Humer E., Kröger I., Reisinger N., Baumgartner W., Wagner M., Zebeli Q. (2020). Starch-rich diet induced rumen acidosis and hindgut dysbiosis in dairy cows of different lactations. Animals (Basel).

[bib30] Pan X.H., Yang L., Beckers Y., Xue F.G., Tang Z.W., Jiang L.S., Xiong B.H. (2017). Thiamine supplementation facilitates thiamine transporter expression in the rumen epithelium and attenuates high-grain-induced inflammation in low-yielding dairy cows. J. Dairy Sci..

[bib31] Pederzolli R.A.A., Van Kessel A.G., Campbell J., Hendrick S., Wood K.M., Penner G.B. (2018). Effect of ruminal acidosis and short-term low feed intake on indicators of gastrointestinal barrier function in Holstein steers. J. Anim. Sci..

[bib32] Peng L., Li Z.R., Green R.S., Holzman I.R., Lin J. (2009). Butyrate enhances the intestinal barrier by facilitating tight junction assembly via activation of AMP-activated protein kinase in Caco-2 cell monolayers. J. Nutr..

[bib33] Plaizier J.C., Li S., Tun H.M., Khafipour E. (2017). Nutritional models of experimentally-induced subacute ruminal acidosis (SARA) differ in their impact on rumen and hindgut bacterial communities in dairy cows. Front. Microbiol..

[bib34] Plöger S., Stumpff F., Penner G.B., Schulzke J.-D., Gäbel G., Martens H., Shen Z., Günzel D., Aschenbach J.R. (2012). Microbial butyrate and its role for barrier function in the gastrointestinal tract. Ann. N. Y. Acad. Sci..

[bib35] Salfer I.J., Morelli M.C., Ying Y., Allen M.S., Harvatine K.J. (2018). The effects of source and concentration of dietary fiber, starch, and fatty acids on the daily patterns of feed intake, rumination, and rumen pH in dairy cows. J. Dairy Sci..

[bib36] Sánchez-Duarte J.I., Kalscheur K.F., Casper D.P., García A.D. (2019). Performance of dairy cows fed diets formulated at 2 starch concentrations with either canola meal or soybean meal as the protein supplement. J. Dairy Sci..

[bib37] Singh N., Gurav A., Sivaprakasam S., Brady E., Padia R., Shi H., Thangaraju M., Prasad P.D., Manicassamy S., Munn D.H., Lee J.R., Offermanns S., Ganapathy V. (2014). Activation of Gpr109a, receptor for niacin and the commensal metabolite butyrate, suppresses colonic inflammation and carcinogenesis. Immunity.

[bib38] Steele M.A., Croom J., Kahler M., AlZahal O., Hook S.E., Plaizier K., McBride B.W. (2011). Bovine rumen epithelium undergoes rapid structural adaptations during grain-induced subacute ruminal acidosis. Am. J. Physiol. Regul. Integr. Comp. Physiol..

[bib39] Steele M.A., Penner G.B., Chaucheyras-Durand F., Guan L.L. (2016). Development and physiology of the rumen and the lower gut: Targets for improving gut health. J. Dairy Sci..

[bib40] Tayyab U., Sinclair L.A., Wilkinson R.G., Humphries D.J., Reynolds C.K. (2022). Milk production, rumen function, and digestion in dairy cows fed diets differing in predominant forage and concentrate type. Anim. Feed Sci. Technol..

[bib41] van Gastelen S., Dijkstra J., Alferink S.J.J., Binnendijk G., Nichols K., Zandstra T., Bannink A. (2021). Abomasal infusion of corn starch and β-hydroxybutyrate in early-lactation Holstein-Friesian dairy cows to induce hindgut and metabolic acidosis. J. Dairy Sci..

[bib42] van Gastelen S., Dijkstra J., Nichols K., Bannink A. (2021). Abomasal infusion of ground corn and ammonium chloride in early-lactating Holstein-Friesian dairy cows to induce hindgut and metabolic acidosis. J. Dairy Sci..

[bib43] Yan H., Ajuwon K.M. (2017). Butyrate modifies intestinal barrier function in IPEC-J2 cells through a selective upregulation of tight junction proteins and activation of the Akt signaling pathway. PLoS One.

[bib44] Yasui T., McCarthy M.M., Ryan C.M., Gilbert R.O., Felippe M.J.B., Mechor G.D., Overton T.R. (2016). Effects of monensin and starch level in early lactation diets on indices of immune function in dairy cows. J. Dairy Sci..

[bib45] Zebeli Q., Ametaj B.N. (2009). Relationships between rumen lipopolysaccharide and mediators of inflammatory response with milk fat production and efficiency in dairy cows. J. Dairy Sci..

[bib46] Zebeli Q., Metzler-Zebeli B.U., Ametaj B.N. (2012). Meta-analysis reveals threshold level of rapidly fermentable dietary concentrate that triggers systemic inflammation in cattle. J. Dairy Sci..

